# NUTRITION ASSESSMENT IN HIGH-RISK PATIENTS: NRS-2002, PG-SGA, AND NFPE IN OLDER ADULTS PREPARING FOR ELECTIVE SURGERY

**DOI:** 10.1093/geroni/igz038.1442

**Published:** 2019-11-08

**Authors:** Kathryn N Porter Starr, Kenlyn Young, Shelley R McDonald, Nancy Loyack, Sandhya ​Sandhya Lagoo-Deenadayalan, Mitchell T Heflin, Carl F Pieper, Connie W Bales

**Affiliations:** 1 Duke University School of Medicine, Durham, North Carolina, United States; 2 Durham VA Medical Center, Durham, North Carolina, United States; 3 Duke University Medical Center, Durham, North Carolina, United States

## Abstract

Post-surgical complications are most common in older adults. While a number of factors contribute, one key determinant is malnutrition. Malnutrition is seen in up to 86% of older adults at hospital admission. Malnutrition and post-surgical complications are linked through two critical observations: 1) malnutrition dramatically reduces the ability of older adults to overcome postsurgical health stressors, and 2) nutritional status is likely to deteriorate further during hospitalization and after discharge. Despite convincing evidence that perioperative nutrition intervention can improve surgical outcomes, nutrition screening and assessment in the preoperative period is not required or standardized. We will review issues surrounding screening and assessment of malnutrition in older adults preparing for elective surgery and present data on screening (NRS-2002) and assessment tools (Nutrition Focused Physical Exam and PG-SGA) used in this high-risk population. Finally, we will discuss best practices for identifying and intervening with malnourished older adults in the preoperative setting.

